# Neurogenic Small Molecules Reverse miR-342-3p-Mediated Tumorigenesis in Renal Cell Carcinoma

**DOI:** 10.14740/wjon2753

**Published:** 2026-06-05

**Authors:** Yi Zhou Ye, Zhang Ming Du, Hong Wei Chen, Qian Xu

**Affiliations:** a Department of Urology, Hangzhou First People’s Hospital Tonglu Hospital, Tonglu County, Hangzhou, Zhejiang, 311500, China; b Department of Obstetrics and Gynecology, Hangzhou First People’s Hospital Tonglu Hospital, Tonglu County, Hangzhou, Zhejiang, 311500, China

**Keywords:** microRNA, Kidney cancer, Cigarette smoking, Lipid metabolism, Neurogenesis

## Abstract

**Background:**

Cigarette smoking is a major established risk factor for clear cell renal cell carcinoma (ccRCC), yet the molecular mediators linking smoking exposure to tumor biology remain incompletely understood. Here, we investigated whether smoking status influences circulating and tissue miR-342-3p and miR-342-5p expression.

**Methods:**

We determined miR-342-3p and miR-342-5p expression levels in tissues and plasmas from ccRCC patients and healthy controls using quantitative reverse transcription polymerase chain reaction. To elucidate the functional relevance of miR-342-3p dysregulation in ccRCC, we integrated miRTARGET and DAVID Gene Ontology analyses to identify ccRCC-related and experimentally validated targets. Cell counting kit-8 assay measured the impact of miR-342-3p mimic and neurogenic small molecules on 293T and 786-O cells.

**Results:**

We found that both miR-342-3p and miR-342-5p were significantly upregulated in ccRCC, with miR-342-3p expression showing a strong positive association with smoking status and highest levels observed in current smokers. Receiver operating characteristic analysis demonstrated that combined plasma miR-342-3p and miR-342-5p expression achieved an area under the curve (AUC) of 0.767, with a sensitivity of 81.6% and a specificity of 69.4%. A total of 178 miR-342-3p ccRCC targets were mainly enriched in lipid metabolic and neurogenesis processes. miR-342-3p overexpression significantly enhanced 293T cell proliferation. However, treatment with a neurogenic small-molecule cocktail (SB431542, LDN193189, CHIR99021, and DAPT) markedly attenuated this proliferative effect. In RCC 786-O cells, the same small molecules significantly inhibited cell proliferation, whereas miR-342-3p overexpression reversed their inhibitory effect.

**Conclusion:**

Cigarette smoking upregulates miR-342-3p and miR-342-5p expression in ccRCC. Mechanistically, miR-342-3p appears to promote RCC tumorigenesis through repression of neurogenic genes. Neurogenic small molecules may confer therapeutic benefit by antagonizing this effect and thereby suppressing RCC progression.

## Introduction

Renal cell carcinoma (RCC) is the most common form of kidney cancer, with its incidence continuing to rise globally. Cigarette smoking is a major established risk factor for RCC incidence and progression [1–3]. Approximately half of RCC patients are current or former cigarette smokers at diagnosis [4, 5]. Unsurprisingly, smoking independently predicts poorer overall survival across all stages of RCC [5]. Quitting smoking can significantly improve RCC patient survival and lower the risk of disease progression [5]. Hence, targeting smoking-related genes could be a promising therapeutic strategy for RCC patients.

MicroRNAs (miRNAs) are a class of small noncoding RNAs, which typically suppress gene translation or promote messenger RNA (mRNA) degradation, thereby controlling a wide range of cellular processes [6]. The dysregulation of specific miRNAs is a recognized feature across many cancer types, including RCC [7, 8]. Growing evidence has demonstrated that miRNAs are often influenced by environmental factors (reviewed in [9]). Multiple studies show that cigarette smoking alters circulating and exosomal microRNA profiles [10–13]. Notably, circulating miR-342-3p (previously known as miR-342) and miR-342-5p are significantly increased in smokers as compared to never smokers [10, 13]. In clear cell RCC (ccRCC), the most prevalent RCC subtype, microarray-based miRNA profiling shows both miR-342-3p and miR-342-5p are markedly upregulated in tumor tissues [8]. Analyses of The Cancer Genome Atlas (TCGA) datasets further indicate that higher expression of miR-342-3p and miR-342-5p correlates with shorter overall survival in RCC patients [14–16], underscoring their potential prognostic value. Moreover, extracellular vesicle–derived miR-342-3p from M2 macrophages has been shown to promote the malignant behavior of RCC cells [17]. Therefore, these findings suggest that smoking-associated miR-342-3p and miR-342-5p may represent promising therapeutic targets in RCC. However, there is no research validating the increased miR-342-3p and miR-342-5p expression in ccRCC. Direct evidence linking patients’ smoking status to miR-342-3p and miR-342-5p expression in RCC is lacking, and the mechanisms by which these miRNAs contribute to RCC progression remain poorly defined.

Cellular reprogramming is an emerging therapeutic approach for cancers [18–21]. Especially, glioma cells can be converted into neuron-like cells by neurogenic transcription factors or certain small molecules [19–21]. In this study, we first identified that plasma and tissue levels of miR-342-3p and miR-342-5p in smoker ccRCC patients and never smoker ccRCC patients were significantly increased compared with healthy controls using quantitative reverse transcription polymerase chain reaction (qRT-PCR). Moreover, we found that miR-342-3p and miR-342-5p expressions were positively associated with smoking status in ccRCC. By conducting Gene Ontology (GO) analyses of ccRCC-related targets of miR-342-3p, we revealed that miR-342-3p contributed to ccRCC development through regulating lipid metabolic processes and neurogenesis. We experimentally demonstrated that small neurogenic molecules could hinder miR-342-3p-mediated RCC development. Taken together, our study suggests small neurogenic molecules may be a potential approach to treat smoking-related ccRCC.

## Materials and Methods

### Study samples

After excluding patients without the pathologic diagnosis and without smoking history data, 85 paired tumor and adjacent non-tumorous renal tissue samples were obtained from patients diagnosed with ccRCC who underwent nephrectomy at Hangzhou First People’s Hospital Tonglu Hospital from 2018 to 2024. Nonfasting blood specimens from 49 ccRCC patients and 38 healthy individuals were collected with EDTA-K2 tubes before any cancer therapy. Blood samples were first centrifuged at 800 *g* for 10 min at 4 °C, and then the supernatant was centrifuged again at 12,000 *g* for 15 min to remove cellular debris. The supernatant was collected as plasma and stored at −80 °C until use [22]. Patients who were actively smoking or had discontinued smoking less than 1 year before surgery were combined into the “current smoker” category for subsequent analysis [3]. Clinical characteristics, specifically smoking status (never-smoker, former smoker, or current smoker), tumor stage, and grade, were retrospectively collected from patient records (Table 1).

**Table 1:** Clinical Characteristics of ccRCC Patients

Characteristic	Current smokers		Former smokers		Never smokers	
	Tissue	Plasma	Tissue	Plasma	Tissue	Plasma
Age, years (mean ± SD)	58 ± 11	57 ± 13	61 ± 15	59 ± 12	62 ± 15	60 ± 13
Gender, male/female	24/10	15/7	18/7	10/7	16/10	5/5
Tumor size, cm (mean ± SD)	7.1 ± 2.2	6.9 ± 3.1	7.2 ± 2.5	7.0 ± 2.8	6.2 ± 1.7	6.3 ± 2.1
T stage, pT1	16	14	14	10	17	6
T stage, pT2	15	7	10	6	8	3
T stage, pT3	2	1	1	1	1	1
T stage, pT4	1	0	0	0	0	0
N stage, N0	32	21	24	17	26	10
N stage, N1	2	1	1	0	0	0
M stage, M0	33	22	25	17	26	10
M stage, M1	1	0	0	0	0	0
Fuhrman grade 1	10	9	6	5	10	3
Fuhrman grade 2	17	11	13	8	11	6
Fuhrman grade 3	5	1	4	2	4	1
Fuhrman grade 4	2	1	2	2	1	0

ccRCC: clear cell renal cell carcinoma; SD: standard deviation.

### Ethical approval

The research related to human use has been complied with all the relevant national regulations, institutional policies and in accordance with the tenets of the Helsinki Declaration and has been approved by the Ethics Committees of Hangzhou First People’s Hospital Tonglu Hospital (No. IRB-20267).

### Tissue RNA extraction

Total tissue RNA was extracted using TRIzol (Thermo Fisher Scientific, Carlsbad, USA) according to the manufacturer’s instructions. Briefly, approximately 50–100 mg of tissue was homogenized in 1 mL of TRIzol using a mechanical homogenizer. After incubation at room temperature for 5 min, 200 µL of chloroform was added, and samples were vigorously mixed and centrifuged at 12,000 *g* for 15 min at 4 °C. The aqueous phase was transferred to a fresh tube, and RNA was precipitated with 500 µL of isopropanol at room temperature for 10 min, followed by centrifugation at 12,000 *g* for 10 min at 4 °C. The RNA pellet was washed with 1 mL of 75% ethanol, air-dried briefly, and resuspended in RNase-free water. The quantification and RNA-quality determination was determined with Nanodrop 8000 (Thermo Scientific). All samples with A260/280 > 1.8 were stored at −80 °C until further analysis.

### Plasma miRNA extraction

Plasma miRNA was extracted in accordance with the manufacturer’s protocol for miRNeasy Serum/Plasma kit (Qiagen, Hilden, Germany) [23]. Briefly, 200 µL of plasma was mixed with 5 volumes of QIAzol lysis Reagent and incubated at room temperature for 5 min. After incubation, 5 pM of cel-miR-39-3p (Qiagen, Hilden, Germany) was spiked into the lysate as a control. Then, 200 µL of chloroform was added, followed by a 2 min incubation at room temperature. The mixture was centrifuged at 12,000 *g* for 15 min at 4 °C to induce phase separation. The upper aqueous phase was carefully transferred to a new microcentrifuge tube and mixed with 1.5 volumes of 100% ethanol. The solution was loaded onto a RNeasy MinElute spin column and centrifuged at 8,000 *g* for 15 s at room temperature. The column was washed sequentially with the RWT and RPE buffers, followed by 80% ethanol. miRNA was resuspended in 15 µL of RNase-free water. The quantification and RNA-quality determination was determined with Nanodrop 8000 (Thermo Scientific). All samples were stored at −80 °C until further analysis.

### qRT-PCR

Reverse transcription was carried out using 0.5 µg of tissue RNA or 4 µL of plasma miRNA, with the RevertAid™ First Strand cDNA Synthesis Kit (Thermo Fisher Scientific, Vilnius, Lithuania) and a reverse transcription primer from Guangzhou RiboBio Co., Ltd. (Guangzhou, China). Then, the diluted cDNA with RNase-free water was mixed with Platinum™ SYBR™ Green qPCR SuperMix-UDG (Thermo Fisher Scientific, Carlsbad, USA) and primers synthesized by Guangzhou RiboBio Co., Ltd. (Guangzhou, China) for qRT-PCR analysis. Mature miRNAs were quantified on a LightCycler^®^ 480 II system (Roche Diagnostics, Basel, Switzerland). The cycling conditions were as follows: 95 °C for 10 min, followed by 40 cycles of 95 °C for 10 s, 60 °C for 20 s, and 72 °C for 1 s. Tissue samples were normalized to U6, and plasma samples were normalized to cel-miR-39. Relative miRNA expression was calculated using the 2^−ΔΔCq^ method.

### Bioinformatics analysis

The miRTARGET web tool [24] was used to identify ccRCC-related targets of miR-342-3p. GO enrichment analysis was conducted using the DAVID database [25].

### Cell culture, transfection, and small molecule treatments

Human embryonic kidney cell line 293T (CRL-3216) and RCC cell line 786-O (CRL-1932) were from ATCC (Manassas, VA, USA). The 293T cells were cultured in medium containing Dulbecco’s modified eagle medium (DMEM, Gibco), 10% fetal bovine serum (FBS, Gibco), and 1% penicillin-streptomycin (Gibco), while 786-O cells were cultured using RPMI-1640 medium (Gibco) with 10% FBS and 1% penicillin-streptomycin. Cells were incubated at 37 °C in a humidified 5% CO_2_ atmosphere. miR-343-3p overexpression was performed through transfection with siRNA/miRNA Transfection Reagent (Cat# HY-K2017, MedChemExpress, Monmouth Junction, NJ, USA) and 50 nM of has-miR-342-3p mimic (Cat# HY-R00708, MedChemExpress) or miRNA mimic negative control (NC mimic) (Cat# HY-R04602, MedChemExpress) in six-well plates. For small molecule treatments, 5 µM SB431542 (Sigma), 0.25 µM LDN193189 (Sigma), 1.5 µM CHIR99021 (Sigma), and 5 µM DAPT (Sigma) were added on the cells cultured with neuronal differential medium including DMEM/F12, 0.5% FBS, 0.8% N2 (Gibco), 1% B27 (Gibco), 20 ng/mL BDNF (Invitrogen), 10 ng/mL GDNF (Invitrogen), 10 ng/mL NT3 (Gibco), and 1 µM Y27632 (Tocris) [19, 26, 27].

### Statistical analysis

All data are presented as mean ± standard deviation (SD). Statistical significance was assessed using Student’s *t*-test, Mann–Whitney U test, or one-way analysis of variance (ANOVA) followed by Bonferroni’s multiple comparisons test, as appropriate. Receiver operating characteristic (ROC) analyses were conducted to assess the diagnostic value of the miRNAs. All statistical analyses were performed using GraphPad Prism 10. A P value < 0.05 was considered statistically significant.

## Results

### Upregulation of tissue miR-342-3p and miR-342-5p in ccRCC patients

**Figure 1:**
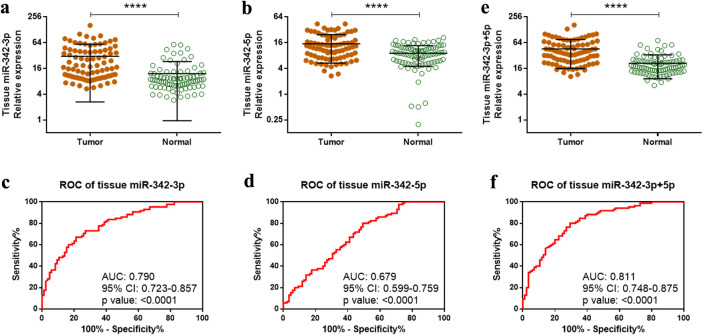
Overexpression of miR-342-3p and miR-342-5p in ccRCC tumor tissues. (a, b) Relative expression of miR-342-3p (a) and miR-342-5p (b) in 85 paired ccRCC tumor and adjacent normal tissues measured by qRT-PCR. (c, d) Diagnostic performance of tissue miR-342-3p (c) and miR-342-5p (d) assessed by ROC analysis. (e) Combined expression of miR-342-3p and miR-342-5p (miR-342-3p+5p) in tumor versus normal tissues. (f) ROC analysis of combined miR-342-3p+5p expression for ccRCC diagnosis. ****P < 0.0001. ccRCC: clear cell renal cell carcinoma; qRT-PCR: quantitative reverse transcription polymerase chain reaction; ROC: receiver operating characteristic.

To validate the previously published microarray findings [8], we quantified the expression of tissue miR-342-3p and miR-342-5p in 85 paired ccRCC tumors and their adjacent normal renal tissues qRT-PCR. Both miRNAs were significantly upregulated in ccRCC tissues compared with normal tissues (P < 0.0001, Fig. 1a, b). To assess their diagnostic potential, we performed ROC analyses. miR-342-3p and miR-342-5p discriminated ccRCC tumor tissues from normal tissues with area under the curve (AUC) values of 0.790 (P < 0.0001) and 0.679 (P < 0.0001), respectively (Fig. 1c, d). miR-342-3p yielded a sensitivity of 72.9% and a specificity of 72.9%, while miR-342-5p showed a sensitivity of 80.0% and a specificity of 50.6%.

Given that these two miRNAs originate from the same precursor, we next evaluated whether their combined abundance (miR-342-3p + miR-342-5p; hereafter referred to as miR-342-3p+5p) offered improved diagnostic performance. Combined expression was significantly higher in ccRCC tumors than in normal tissues (Fig. 1e). The ROC curve for the combined metric demonstrated an AUC of 0.811 (P < 0.0001), with a sensitivity of 80.0% and a specificity of 70.1% (Fig. 1f).

### Upregulation of circulating miR-342-3p and miR-342-5p in ccRCC patients

**Figure 2:**
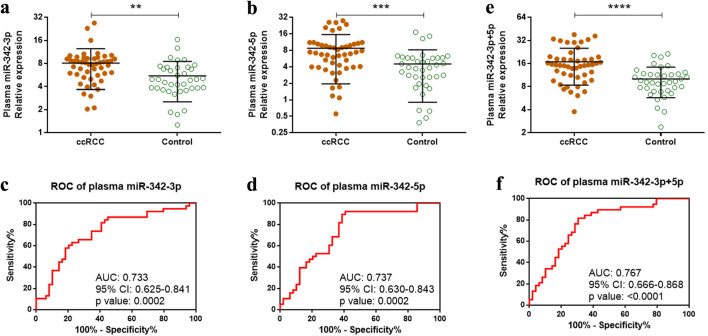
Overexpression of plasma miR-342-3p and miR-342-5p in ccRCC patients. (a, b) Relative plasma expression of miR-342-3p (a) and miR-342-5p (b) in 49 ccRCC patients versus 38 healthy controls measured by qRT-PCR. (c, d) Diagnostic performance of plasma miR-342-3p (c) and miR-342-5p (d) assessed by ROC analysis. (e) Combined plasma expression of miR-342-3p and miR-342-5p (miR-342-3p+5p) in ccRCC patients versus controls. (f) ROC analysis of combined plasma miR-342-3p+5p for ccRCC detection. **P < 0.01; ***P < 0.001; ****P < 0.0001. ccRCC: clear cell renal cell carcinoma; qRT-PCR: quantitative reverse transcription polymerase chain reaction; ROC: receiver operating characteristic.

Circulating miRNAs are emerging as promising biomarkers that may enhance early detection, reduce invasive procedures, and improve therapeutic management and follow-up [28]. Based on this rationale, we quantified plasma miR-342-3p and miR-342-5p levels in 49 ccRCC patients and 38 healthy controls. Both miRNAs were significantly elevated in the plasma of ccRCC patients compared with healthy individuals (Fig. 2a, b). ROC analysis demonstrated that plasma miR-342-3p and miR-342-5p could effectively discriminate ccRCC patients from healthy controls, with AUC values of 0.733 (P = 0.0002) and 0.737 (P = 0.0002), respectively (Fig. 2c, d). Plasma miR-342-3p yielded a sensitivity of 86.8% and a specificity of 55.1%, whereas miR-342-5p achieved a sensitivity of 92.1% and a specificity of 59.2%. The combined plasma expression of miR-342-3p+5p was also markedly elevated in ccRCC patients relative to healthy controls (Fig. 2e). The combined ROC analysis produced an AUC of 0.767 (P < 0.0001), with a sensitivity of 81.6% and a specificity of 69.4% (Fig. 2f). Collectively, these findings support plasma miR-342-3p and miR-342-5p as promising noninvasive biomarkers for ccRCC detection.

### miR-342-3p and miR-342-5p positively correlate with smoking status

**Figure 3:**
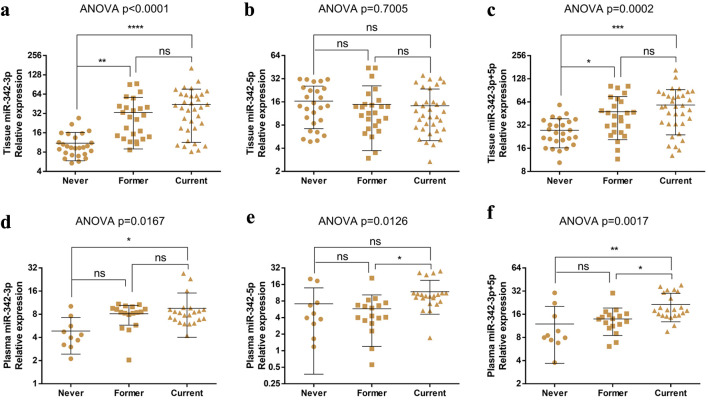
miR-342-3p expression levels are related to the smoking status of ccRCC patients. (a–c) Tissue levels of miR-342-3p (a), miR-342-5p (b), and combined miR-342-3p+5p (c) across smoking-status groups with ccRCC. (d–f) Plasma levels of miR-342-3p (d), miR-342-5p (e), and combined miR-342-3p+5p (f) across smoking-status groups with ccRCC. ns: no significant change; *P < 0.05; **P < 0.01; ***P < 0.001; ****P < 0.0001. ccRCC: clear cell renal cell carcinoma.

To determine whether miR-342-3p and miR-342-5p are associated with cigarette smoking status in ccRCC patients, we analyzed their expression in tissue and plasma samples stratified by smoking category (never smokers, former smokers, and current smokers). One-way ANOVA followed by Bonferroni’s multiple comparison tests was performed for miR-342-3p, miR-342-5p, and their combined abundance. ANOVA revealed significant overall differences for all comparisons (Fig. 3), except for tissue miR-342-5p (Fig. 3b). Bonferroni’s *post hoc* tests showed that tissue miR-342-3p levels were significantly higher in both former and current smokers than in never smokers (Fig. 3a), with no significant difference between former and current smokers. The combined tissue miR-342-3p and miR-342-5p metric produced a similar pattern (Fig. 3c). Notably, miR-342-3p expression levels or the combined levels of miR-342-3p and miR-342-5p displayed an upward trend in current smokers relative to former smokers, although this did not reach statistical significance (Fig. 3a, c).

In plasma, miR-342-3p expression was significantly elevated in current smokers compared with never smokers, but not former smokers (Fig. 3d). Plasma miR-342-5p levels were higher in current smokers than in former smokers (Fig. 3e), although no significant differences were observed between never smokers and the other groups. The combined plasma miR-342-3p and miR-342-5p expression was markedly elevated in current smokers compared with both never and former smokers, with no significant difference between never and former smokers (Fig. 3f).

Together, these results indicate that miR-342-3p levels are positively associated with smoking status in ccRCC patients, with the highest expression observed in current smokers.

### miR-342-3p is associated with poor overall survival

Given that smoking is an independent predictor of poorer overall survival across all stages of RCC [5] and miR-342-3p was significantly overexpressed in smokers (Fig. 3), we next investigated whether miR-342-3p possesses prognostic value in ccRCC. Because miRNAs exert their regulatory effects by binding to complementary sequences in the 3′-untranslated regions (3′-UTRs) of target mRNAs, we sought to identify downstream targets that might mediate this effect. We utilized the miRTARGET web tool [24] to identify ccRCC-related targets of miR-342-3p based on the following stringent criteria: 1) upregulated by more than 1.25-fold in miR-342-3p knockout/knockdown models in at least 40% of studies; 2) negatively correlated with miR-342-3p expression (correlation coefficient ≤ −0.1) in at least 20% of cancer types; 3) significantly downregulated in ccRCC (called KIRC, clear cell renal carcinoma, in this web tool); 4) high target expression associated with favorable overall survival in ccRCC; and 5) predicted by at least two independent miRNA target-prediction algorithms. miRTARGET extracts data from TCGA to identify cancer-related targets [24].

**Figure 4:**
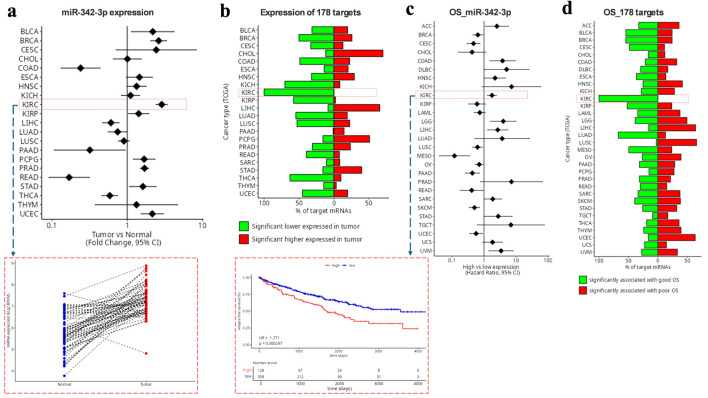
Identification of miR-342-3p targets in ccRCC using miRTarget. (a) miR-342-3p expression in tumor versus normal tissues across the indicated cancer types. The red rectangle highlights miR-342-3p expression in KIRC. (b) Proportion of the 178 putative KIRC-related miR-342-3p targets that are upregulated or downregulated in tumors compared with normal tissues. (c) Associations between miR-342-3p expression levels and OS across the indicated cancer types. The red rectangle highlights the KIRC survival curve. (d) Proportion of the 178 targets associated with favorable or poor OS in KIRC. ccRCC: clear cell renal cell carcinoma; KIRC: clear cell renal carcinoma; OS: overall survival.

Indeed, miRTARGET shows that miR-342-3p expression was elevated across multiple tumor types, including ccRCC (Fig. 4a). Using the above parameters, we identified 178 high-confidence miR-342-3p targets associated with ccRCC, which were significantly downregulated in ccRCC tissues (Fig. 4b). High miR-342-3p expression was associated with poorer overall survival in ccRCC patients (P = 0.0003; Fig. 4c), while reduced expression of its target set correlated with improved survival (Fig. 4d).

Collectively, these findings indicate that elevated miR-342-3p expression is associated with adverse overall survival outcomes in ccRCC.

### miR-342-3p ccRCC targets are enriched in lipid metabolic processes and neurogenesis

**Figure 5:**
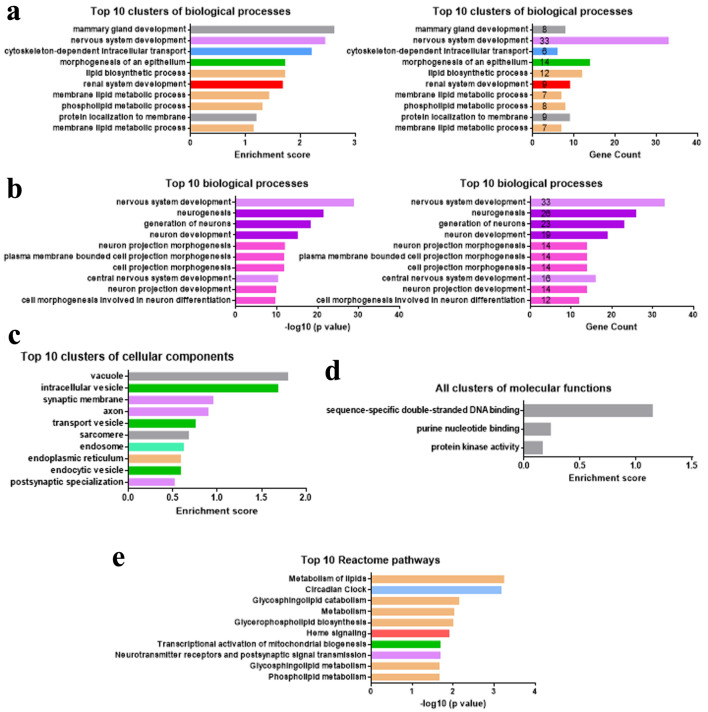
miR-342-3p regulates genes involved in lipid metabolism and neurogenesis. (a–d) Top 10 or all enriched biological process (a, b), cellular component (c), or molecular function (d) clusters among 178 miR-342-3p ccRCC-related target genes. (e) Top 10 Reactome pathways in 178 miR-342-3p ccRCC-related targets. ccRCC: clear cell renal cell carcinoma.

We next investigated the major functional categories of miR-342-3p ccRCC targets using GO enrichment analysis in the DAVID database [25]. The top 10 enriched biological process (BP) clusters indicated that miR-342-3p targets were strongly associated with lipid metabolic processes (Fig. 5a). Additional enriched clusters involved mammary gland, nervous, and renal system development, cytoskeletal organization, and epithelial morphogenesis. Lipid-related targets included CDS1, CLN8, ASAH1, PBX1, ETNK2, GGT6, PIGH, PGAP3, PPM1L, SLC44A3, SLC44A4, and TMEM68, whereas renal-related targets comprised FRAS1, KLF15, MTSS1, PBX1, COL4A4, ERBB4, OVOL1, PTCH1, and PRKX. Notably, nervous system development exhibited the highest gene count among these BP clusters and ranked second by enrichment score (Fig. 5a). To further understand the function of the 33-nervous related targets, we performed GO analysis for them. As shown in Figure 5b, the top 10 enriched BPs were mainly neurogenesis and neuron morphogenesis-related. Of the nervous system targets, 78.8% (26/33) were neurogenesis-related.

The top 10 enriched cellular component clusters suggested that miR-342-3p targets were mainly in the membrane-bound sacs (intracellular vesicles, transport vesicles, endocytic vesicles), endosome, and endoplasmic reticulum, which are responsible for transporting substances including lipids (Fig. 5c). Synapse and axon-related cellular components were also enriched in the 178 miR-342-3p ccRCC targets. The molecular functions of miR-342-3p ccRCC targets were related to DNA binding and protein kinase activity (Fig. 5d). Most Reactome pathways of these targets were also lipid metabolism-related (Fig. 5e).

Therefore, our GO analyses of 178 miR-342-3p ccRCC targets revealed that miR-342-3p might regulate lipid metabolic processes and neurogenesis during ccRCC carcinogenesis and development.

### Small neurogenic molecules inhibit RCC development

**Figure 6:**
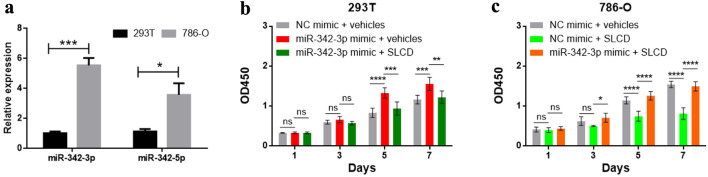
Quantitative analysis of cell proliferation by CCK-8 assay. (a) miR-342-3p and miR-342-5p expression levels in 293T and 786-O cells determined by qRT-PCR. (b, c) The cell proliferation was measured by CCK-8. ns: not significant; *P < 0.05; **P < 0.01; ***P < 0.001; ****P < 0.0001. CCK-8: cell counting kit-8; qRT-PCR: quantitative reverse transcription polymerase chain reaction.

Recent studies reveal direct neuronal reprogramming as a novel approach for glioma treatment through converting proliferative cancer cells into non-dividing neurons with small molecules or neurogenic transcription factors [19, 20, 26, 29, 30]. Based on our above findings, we reasoned that those small molecules and neurogenic factors could inhibit miR-342-3p-mediated ccRCC development. To test this hypothesis, we first confirmed the downregulated expression levels of 26 neurogenesis-related targets of miR-342-3p in KIRC (Table 2) using OncoDB 2.0 [31] and overexpression levels of miR-342-3p and miR-342-5p in 786-O RCC cells compared with human embryonic kidney 293T cells (Fig. 6a). We then examined if miR-342-3p overexpression enhanced the proliferative ability of 293T cells. Our CCK8 assay showed that miR-342-3p mimic dramatically promoted 293T cell proliferation (Fig. 6b). Four small molecules (SB431542, LDN193189, CHIR99021, and DAPT) have been recently demonstrated to be necessary for converting glioma cells into neuronal-like cells [19, 26]. By adding the small molecule cocktail SLCD (SB431542, LDN193189, CHIR99021, and DAPT) of direct neuronal reprogramming, the proliferative promoting effect of miR-342-3p was greatly inhibited (Fig. 6b). To further demonstrate the inhibitory effect of the small neurogenic molecules on RCC, we treated 786-O RCC cells with these small molecules. Expectedly, 786-O cell proliferation was significantly inhibited by these small molecules (Fig. 6c). Overexpression of miR-342-3p on 786-O cells diminished the beneficial effect of small molecules (Fig. 6c). Unfortunately, we did not observe the significant changes in neuronal morphology (neurites) in 293T or 786-O cells treated with SLCD (data not shown).

**Table 2:** FC and P Value of 26 Neurogenesis-Related Targets of miR-342-3p in KIRC as Compared to Normal Kidney Tissues (Extracted From OncoDB 2.0)

Gene	KIRC (n = 545) vs. normal (n = 72)		
	log_2_FC	FC	P value
CHL1	−4.7700	0.0367	2.90 × 10^−30^
ERBB4	−4.1000	0.0583	3.00 × 10^−24^
SEMA6D	−3.4300	0.0928	7.20 × 10^−17^
SLC44A4	−3.3800	0.0961	6.50 × 10^−23^
THRB	−2.4200	0.1869	7.60 × 10^−31^
NTN1	−2.2300	0.2132	6.00 × 10^−2^
FLRT3	−2.0500	0.2415	4.50 × 10^−28^
PBX1	−1.8700	0.2736	1.30 × 10^−35^
NTN4	−1.8300	0.2813	6.70 × 10^−18^
KLF15	−1.6400	0.3209	1.10 × 10^−7^
COBL	−1.5300	0.3463	3.30 × 10^−24^
CTF1	−1.4800	0.3585	4.60 × 10^−22^
MYO6	−1.4600	0.3635	7.70 × 10^−35^
SOX6	−1.4000	0.3789	3.00 × 10^−27^
ATP9A	−1.3400	0.3950	3.40 × 10^−22^
GABRB3	−1.3200	0.4005	1.70 × 10^−8^
LAMC3	−1.1700	0.4444	2.90 × 10^−5^
PTCH1	−1.1500	0.4506	1.00 × 10^−19^
ADCY1	−1.1300	0.4569	2.70 × 10^−5^
FZD3	−1.0400	0.4863	5.30 × 10^−25^
SEMA5A	−1.0300	0.4897	1.80 × 10^−8^
APLP2	−0.8700	0.5471	6.40 × 10^−34^
WASL	−0.7200	0.6071	6.20 × 10^−26^
CLN8	−0.6600	0.6329	4.70 × 10^−7^
ID4	−0.6000	0.6598	8.40 × 10^−6^
PPP1R12B	−0.5900	0.6643	9.80 × 10^−3^

FC: fold change; KIRC: clear cell renal carcinoma.

In summary, our findings suggest that miR-342-3p may promote RCC carcinogenesis through inhibiting neurogenic genes and small neurogenic molecules have the potential to hinder RCC development.

## Discussion

**Figure 7:**
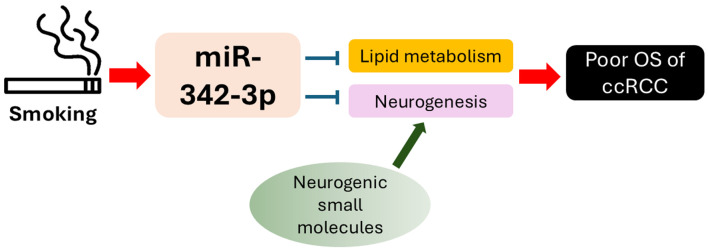
Schematic summarizing the proposed biological functions of miR-342-3p in ccRCC. ccRCC: clear cell renal cell carcinoma.

In this study, we demonstrated that both miR-342-3p and miR-342-5p were significantly upregulated in ccRCC tissues and plasma. Tissue and plasma miR-342-3p levels were positively associated with smoking status, with the highest expression observed in current smokers. For the first time, we found that miR-342-3p mainly influenced lipid metabolism and neurogenesis. Importantly, small neurogenic molecules showed great potential for treating smoking-associated ccRCC (Fig. 7).

Although miR-342 family members were upregulated in ccRCC in our study, they are notably downregulated in several other tumor types, including non-small cell lung cancer (NSCLC) [32], osteosarcoma [33], breast cancer [34], and glioblastoma [35]. Circulating miR-342 is also reduced in the plasma of acute myeloid leukemia patients [36] and in the serum of NSCLC patients [37]. Consequently, miR-342 has historically been regarded as a tumor suppressor [38]. However, TCGA-derived miRNA expression profiling across 21 cancer types reveals that miR-342 is markedly upregulated in more than half (12/21) of epithelial malignancies, including bladder urothelial carcinoma (BLCA), breast carcinoma (BRCA), cervical carcinoma (CESC), ccRCC (KIRC), and uterine corpus endometrial carcinoma (UCEC) (Fig. 4a). Furthermore, miR-342-3p is overexpressed in colon cancer and correlates with worse patient prognosis [39]. These findings indicate that miR-342-3p and miR-342-5p expressions are highly context-dependent and vary substantially across tumor types.

Recent studies have shown that environmental hazards can epigenetically alter cellular and circulating miRNA profiles, underscoring the potential of miRNAs as sensitive biomarkers and therapeutic targets in environmental health [9]. Smoking-related epidemiological evidence provides a plausible link to the elevated miR-342-3p and miR-5p levels in ccRCC. Willinger et al [13] analyzed whole blood–derived RNA from 5,023 participants of the Framingham Heart Study and identified a six-miRNA smoking signature. Interestingly, miR-342-5p was uniquely elevated in both former and current smokers relative to never smokers [13]. More recently, Karabegovic et al [10] reported 41 smoking-associated circulating miRNAs in 2,686 participants from the Rotterdam Study, with 34 miRNAs including miR-342-3p significantly increased in current smokers. Given that cigarette smoking is an established risk factor for RCC, upregulation of miR-342-3p and miR-342-5p in ccRCC is consistent with these observations. Importantly, smoking cessation after RCC diagnosis improves survival and reduces progression risk [40]. Circulating miR-342-3p gradually declines with long-term cessation, showing a significant reduction after more than 15 years [10]. Concordantly, our data revealed decreased plasma miR-342 levels in former smokers, with statistically significant reductions in miR-342-5p and combined miR-342-3p and miR-342-5p.

Functionally, miR-342-3p and miR-342-5p have been reported to inhibit tumor growth and metastasis in several cancer models, suggesting their potential use as therapeutic agents [41–44]. Beyond oncology, miR-342-5p contributes to Alzheimer’s disease–related axonopathy by targeting ankyrin G [45], and protects neurons from ischemia-induced apoptosis through Akt/NF-κB signaling [46]. miR-342-3p is upregulated in microglia following TNF-α stimulation and mediates neurotoxic responses [47]. It is also enriched in adipose tissue and strongly promotes adipogenic differentiation [48]. Consistent with these observations, our GO analysis revealed a strong enrichment of lipid metabolism and neurogenesis among miR-342-3p targets.

Unlike traditional treatments, cancer cell reprogramming, a novel strategy of cancer treatment, does not kill cancer cells and normal cells. Therefore, this approach could mitigate the substantial off-target toxicity associated with conventional chemotherapy and radiotherapy [20, 49]. Converting glioma cells into neuronal-like cells is one example of cancer cell reprogramming. SLCD contributes to neuronal reprogramming through regulating the Notch, TGF-beta, and Wnt signaling pathways [19], which are frequently overactivated in RCC [50, 51]. Targeting these pathways has been shown to inhibit RCC tumor growth [50, 51]. Although the small molecule cocktail SLCD did not convert RCC cells into neuronal-like cells, this cocktail indeed significantly inhibited RCC cell proliferation, suggesting that small neurogenic molecules may partly change RCC cell fate at the molecular level.

### Limitations

Our study has several limitations. First, the sample size was relatively small. Second, *in vivo* functional experiments investigating miR-342-3p were not performed using RCC mouse models. Third, the findings derived from bioinformatics analyses require more experimental validation. In particular, the putative neurogenic targets of miR-342-3p should be validated using luciferase reporter assays to confirm direct binding to the mRNA 3′-UTR, along with rescue experiments to determine whether these targets are functionally relevant. Fourth, the diagnostic ability of plasma miR-342-3p and miR-342-5p for ccRCC was less than 0.8 in our study. Fifth, although we observed a positive association between miR-342-3p levels and smoking status in ccRCC patients, there is currently no direct evidence demonstrating that cigarette smoke extract induces miR-342-3p and miR-342-5p expression in RCC cells or in non-malignant renal epithelial (HK-2) and 293T cell lines. Accordingly, their clinical value needs to be investigated in a large scale of patients with ccRCC. Further mechanistic studies are warranted to elucidate the precise roles of miR-342-3p–mediated pathways in the pathogenesis of ccRCC.

### Conclusion

In conclusion, cigarette smoking induces miR-342-3p and miR-342-5p expression in ccRCC. miR-342-3p may facilitate RCC tumorigenesis by suppressing neurogenic gene expression, whereas small neurogenic molecules may exert therapeutic potential by counteracting this effect and inhibiting RCC progression.

## Data Availability

The data supporting the findings of this study are available from the corresponding author upon reasonable request.

## Abbreviations

3′-UTRs: 3′-untranslated regions
AUC: area under the curve
BLCA: bladder urothelial carcinoma
BRCA: breast carcinoma
ccRCC: clear cell renal cell carcinoma
CESC: cervical carcinoma
DR: downregulation
FC: fold change
GO: Gene Ontology
KIRC: clear cell renal carcinoma
NS: no significant change
NSCLC: non-small cell lung cancer
OS: overall survival
qRT-PCR: quantitative reverse transcription polymerase chain reaction
ROC: receiver operating characteristic
SD: standard deviation
TCGA: The Cancer Genome Atlas
UCEC: uterine corpus endometrial carcinoma
UR: upregulation
